# Tobacco etch virus (TEV) protease with multiple mutations to improve solubility and reduce self‐cleavage exhibits enhanced enzymatic activity

**DOI:** 10.1002/2211-5463.12828

**Published:** 2020-03-18

**Authors:** Heejin Nam, Beom J. Hwang, Deog‐Young Choi, Sooim Shin, Moonsung Choi

**Affiliations:** ^1^ Interdisciplinary Program of Bioenergy and Biomaterials Graduate School College of Engineering Chonnam National University Gwangju South Korea; ^2^ Department of Biotechnology College of Life Science and Biotechnology Yonsei University Seoul South Korea; ^3^ InThera INC Seoul South Korea; ^4^ Department of Bioengineering and Biotechnology College of Engineering Chonnam National University Gwangju South Korea; ^5^ Department of Optometry College of Energy and Biotechnology Seoul National University of Science and Technology South Korea; ^6^ Convergence Institute of Biomaterials and Bioengineering Seoul National University of Science and Technology South Korea

**Keywords:** enzyme kinetics, inclusion body, multiple mutations, self‐inactivation, substrate specificity, tobacco etch virus protease

## Abstract

Tobacco etch virus (TEV) protease is a 27‐kDa catalytic domain of the polyprotein nuclear inclusion a (NIa) in TEV, which recognizes the specific amino acid sequence ENLYFQG/S and cleaves between Q and G/S. Despite its substrate specificity, its use is limited by its autoinactivation through self‐cleavage and poor solubility during purification. It was previously reported that T17S/N68D/I77V mutations improve the solubility and yield of TEV protease and S219 mutations provide protection against self‐cleavage. In this study, we isolated TEV proteases with S219N and S219V mutations in the background of T17S, N68D, and I77V without the inclusion body, and measured their enzyme kinetics. The *k*_cat_ of two isolated S219N and S219V mutants in the background of T17S, N68D, and I77V mutations was highly increased compared to that of the control, and S219N was twofold faster than S219V without *K*_m_ change. This result indicates that combination of these mutations can further enhance TEV activity.

## Graphical abstract

The use of tobacco etch virus (TEV) protease is limited by its poor solubility and self‐inactivation. We analyzed the effect of quadruple mutations (T17S, N68D, I77V, and S219X), which affect solubility and inhibit autoinactivation in TEV protease. In a background of T17S, N68D, and I77V mutations, soluble S219N mutant TEV protease serves as a more efficient catalyst than soluble S219V mutant.

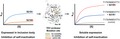

**Abbreviations**
5‐FAM: 5‐carboxyfluorescein
Ex/Em: Excitation over emission wavelength
NaPi: Sodium phosphate
Ni‐NTA: Nickel/nitrilotriacetic acid
Polyprotein Nia: Polyprotein nuclear inclusion a
RFU: Relative fluorescence unit
RID: RNA interaction‐mediated domain
TEV: Tobacco etch virus

Tobacco etch virus (TEV) belongs to the Potyvirus family [1] and forms a cylindrical inclusion body in the cytoplasm of plants [2]. TEV possesses a positive–sense single‐stranded RNA containing approximately 9500 bases, and its RNA genome is translated into a polyprotein called nuclear inclusion a (NIa), which possesses a 27‐kDa catalytic domain with trypsin‐like serine proteolytic activity [3, 4, 5]. The catalytic triad residues of the active site are His46, Asp81, and Cys151, and are located at the interior of the domain (Fig. 1). These residues recognize the amino acid sequence ENLYFQG/S on a target protein and cleave the peptide bond between Q and G/S [6, 7, 8, 9]. Due to this specific function, the TEV protease can be utilized in various protein‐engineering fields. For example, protein–protein interaction can be monitored by the TEV protease using protease fragment complementation and a reporter fusion protein containing the TEV cleavage site [10]. The TEV protease can also be used for multiple gene expression through the expression of a single expression vector encoding the TEV protease, other target proteins, and the TEV substrate sequence [11]. However, there are several limitations when using the TEV protease. One hurdle is its poor solubility during protein expression and purification due to its high hydrophobicity. An inclusion body is easily formed during protein expression [12]; therefore, the protease has to be solubilized and reconstituted to make it active again. These additional purification steps increase the cost of TEV protease. To overcome the limitation of solubility, random and rational mutagenesis and the addition of a combination of several tags, such as glutathione S‐transferase or maltose binding protein, were performed on TEV protease [13]. The T17S/N68D/I77V mutant TEV protease was generated by random mutagenesis, and these mutations improve the solubility and yield of TEV protease effectively because they are located at or near the surface of TEV protease [14]. Additionally, the T17S/N68D/I77V mutant is more stable than the wild‐type protease because the mutations result in more rigid secondary structure elements, such as helices and sheets [15].

**Fig. 1:**
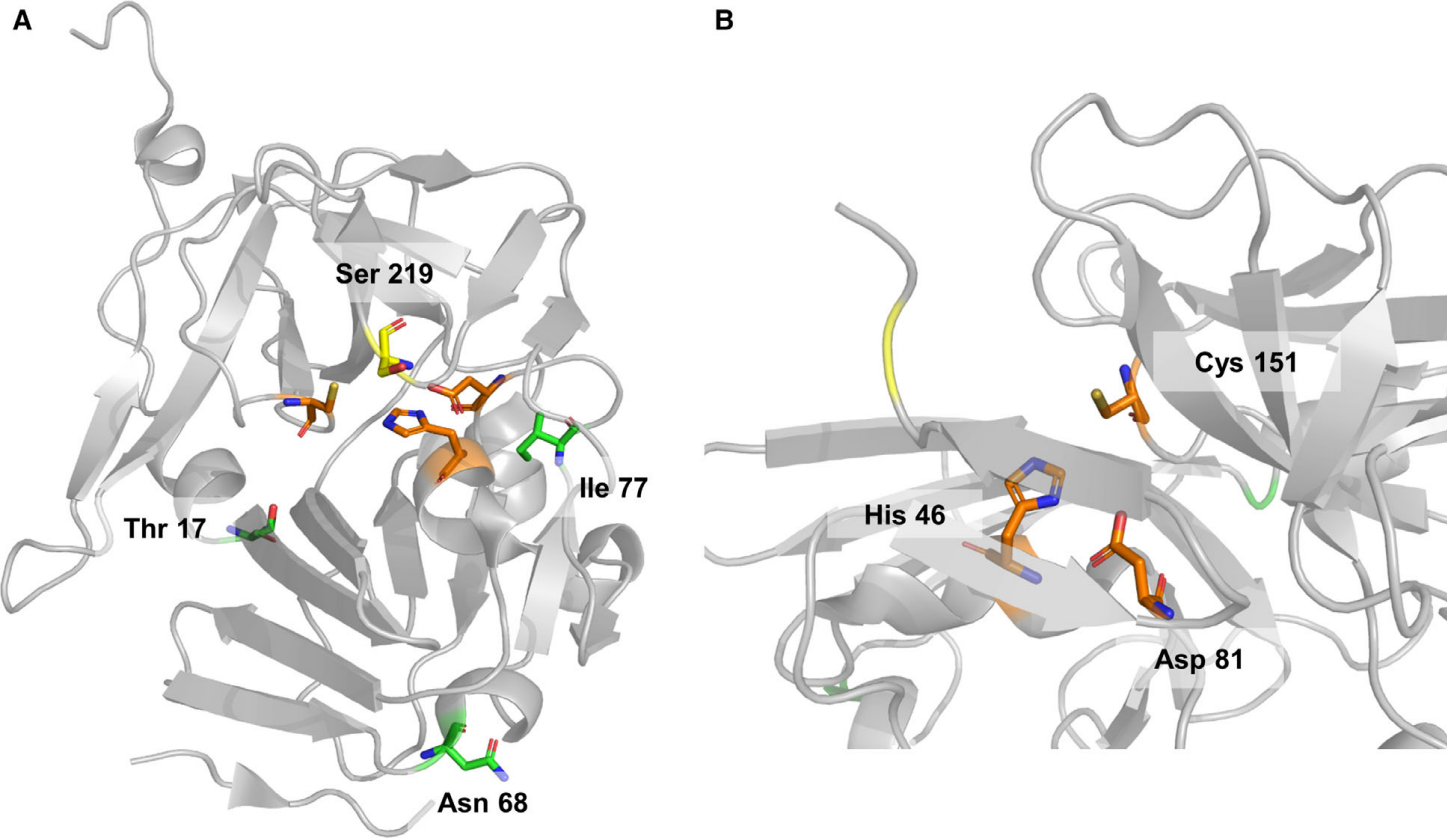
The structure of the catalytic domain of wild‐type TEV protease. (A) Structure of TEV protease. The green residues indicate the background mutations, and the yellow residue is the target residue for mutation, Ser219. The orange residues are the catalytic triad residues, His46, Glu81, and Cys151. (B) The active site of TEV protease, containing the catalytic triad residues. These figures were produced by the pymol program (PyMOL, Schrödinger, NY, USA).

Another limitation is that the wild‐type TEV protease truncates itself during production and purification, decreasing its activity [16]. A cleavage site of TEV protease resides at the C terminus, and the truncated C‐terminal sequences inhibit TEV protease by tightly binding to it [17]. Several studies have shown that Ser219 is a key residue for the self‐cleavage of TEV protease. Thus, this serine residue was substituted with various amino acids in an attempt to inhibit self‐cleavage of TEV protease, and as a result, it was discovered that the S219V mutant was the most efficient at inhibiting the truncation and was more stable than the wild‐type protease [18].

In this study, we tried to introduce two types of mutations into TEV protease simultaneously to improve solubility and to protect against self‐cleavage. The S219 mutation that provides protection against self‐cleavage [18] was introduced into a background of T17S, N68D, and I77V mutations, which increase the solubility and stability of the protease [14, 15], and the enzyme kinetics of both engineered TEV proteases was analyzed and compared.

## Materials and methods

### Cloning the mutant TEV protease gene

The TEV protease gene, containing the mutations S219N, T17S, N68D, and I77V to prevent the autoproteolysis of wild‐type TEV protease and to prevent it from being insoluble, was synthesized by Cosmogenetech (Seoul, Korea) [14]. The synthesized mutant gene was cloned into the pGEMEX‐1 vector (Promega, Madison, WI, USA, discontinued product) using XbaI (New England Biolabs, Ipswich, MA, USA, catalogue no. R0145S) and SalI (New England BioLabs, catalogue no. R3138), and ligated with a T4 ligation kit (Takara, Kusatsu, Shiga, Japan, catalogue no. 6021). The point mutation was generated to replace the S219N mutation with S219V using the Dokdo™ Site‐Specific Mutagenesis Kit (Elpis Biotech, Daejeon, Korea, catalogue no. EBT‐5001). The final expression plasmid was transformed into the *Escherichia coli* strain BL21(DE3)pLysE.

### Mutant TEV protease preparation

Mutant TEV proteases were isolated according to the modified protocol based on the previously reported method [14]. BL21(DE3)pLysE containing the TEV protease gene plasmid was cultured overnight in 4 mL LB media with ampicillin (100 μg·mL^−1^) and chloramphenicol (30 μg·mL^−1^) at 30 °C. All preculture cells were inoculated in 500 mL LB broth with the same antibiotics and cultured at 37 °C until the optical density (OD) at 600 nm reached 0.6. Protein overexpression was induced by the addition of 1 mm IPTG for 20 h at 20 °C. The cells were harvested by centrifugation at 5000 ***g*** for 15 min. The pellet from the culture was resuspended in 50 mL lysis buffer at pH 7.0 consisting of 50 mm sodium phosphate (NaPi) and 300 mm NaCl. For protein extraction, the cells were lysed by sonication using the Branson 450 Digital Sonifier and the lysate was centrifuged at 14 981 ***g*** (14 980 ***g***) for 30 min. The pellet and supernatant were separated into different tubes. The pellet was resuspended in 20 mL Tris‐buffered saline containing 50 mm Tris and 150 mm NaCl at pH 7.6. SDS/PAGE was performed to check the solubility of the expressed protein. Before the sample was loaded to the FPLC, the line of the FPLC and the immobilized metal affinity chromatography column was washed using a buffer containing 50 mm NaPi and 300 mm NaCl at pH 7.0. The soluble fraction, which was separated from the pellet immediately, was run through the FPLC, and the sample was maintained in the cold state using ice due to the protease activity. After the supernatant was loaded onto the nickel/nitrilotriacetic acid (Ni‐NTA) column, the nonspecific binding proteins were eluted by buffer containing 20 mm imidazole with 300 mm NaCl and 50 mm NaPi at pH 7.0. The TEV protease was eluted by buffer containing 250 mm imidazole with 300 mm NaCl and 50 mm NaPi at pH 7.0. Each of the eluted fractions was collected in a different tube according to the buffer, and fractions were collected from sample injection to elution of TEV protease. Next, SDS/PAGE was performed on the fractions to confirm the purity of the protein and to determine the sample for concentration. The protein purity in each fraction was judged by SDS/PAGE, and the pure sample was concentrated with 50 mm NaPi buffer to remove residual salt and imidazole using a 10000‐cut Amicon® Ultra centrifugal filter (Sigma‐Aldrich, St. Louis, MO, USA). After concentration, the OD of the sample at 280 nm was measured by UV/Vis spectrophotometry to calculate the approximate concentration of the protein using an extinction coefficient of 32 220 m ^−1^·cm^−1^ obtained from the ExPASy program. Glycerol was added at a final concentration of 10%, and the sample was stored at –80 °C. The concentration of TEV protease was also confirmed by the Bradford assay with BSA as a standard. BSA was diluted to different concentrations in a range covering the concentration of the sample, and then, Bradford dye was added. After a 5‐min reaction, the OD at 595 nm was measured and plotted against the BSA concentration. TEV protease (1 μL) in the kit was diluted in PBS buffer and measured using the same method used for the standard samples.

### Activity of TEV protease

The SensoLyte® 520 TEV Protease Assay Kit (Anaspec, Fremont, CA, USA) was used to measure the activity of TEV protease. 5‐Carboxyfluorescein (5‐FAM) solution was diluted to 0, 125, 250, and 500 nm in assay buffer containing 1 mm DTT to obtain a standard curve. One hundred microliters of each sample was added to a black 96‐well plate. The intensity of excitation/emission at 485 nm/535 nm was measured, and the relative fluorescence unit (RFU) was plotted along with the 5‐FAM concentration. Steady‐state kinetics was analyzed through the reaction of TEV protease with the 5‐FAM fused substrate. The appearance of 5‐FAM cleaved by TEV protease was monitored, and the initial velocities corresponding to varying concentrations of the substrate were calculated. TEV protease was diluted to 1 μg·mL^−1^, and its substrate (100 μm of stock solution) was diluted to different concentrations. In the black 96‐well plate, 50 μL diluted TEV protease was combined with 50 μL of substrate at varying concentrations. With a total reaction volume of 100 μL, the intensity of 485 nm over 535 nm (Ex/Em) was measured every minute for 2 h and 30 min. Steady‐state kinetic parameters were determined by fitting the data to the Michaelis–Menten equation:

(1) $$V_{0}=\frac{V_{\max}\left[S\right]}{K_{m}+\left[S\right]}$$

(2) $$k_{\text{cat}}=\frac{V_{\max}}{{\left[E\right]}_{t}}$$

*V*_0_ is the initial velocity of the reaction, and [S] is substrate concentration quenching 5‐FAM. *V*_max_ represents the maximum rate achieved by the system at the saturating substrate concentration. The Michaelis constant *K*_m_ is the substrate concentration at which the reaction rate is half of *V*_max_. *k*_cat_ is the turnover number and represents how many substrate molecules are turned over per unit of time by TEV protease. [E]*_t_* represents the total enzyme concentration used.

### TEV protease activity confirmed by SDS/PAGE

TEV protease was mixed with its substrate, the 65‐kDa recombinant RNA interaction‐mediated domain (RID) fused with the norovirus P domain in 50 mm Tris/HCl (pH 8.0), 0.5 mm EDTA, and 1 mm DTT buffer. The reaction solution was collected at 5, 10, 20, 30, 40, 60, 100, and 150 min, and then, the reaction was stopped by boiling at 95 °C. Changes in substrate cleavage were assessed over time by 12% SDS/PAGE.

## Results

### Protein expression and purification

**Fig. 2:**
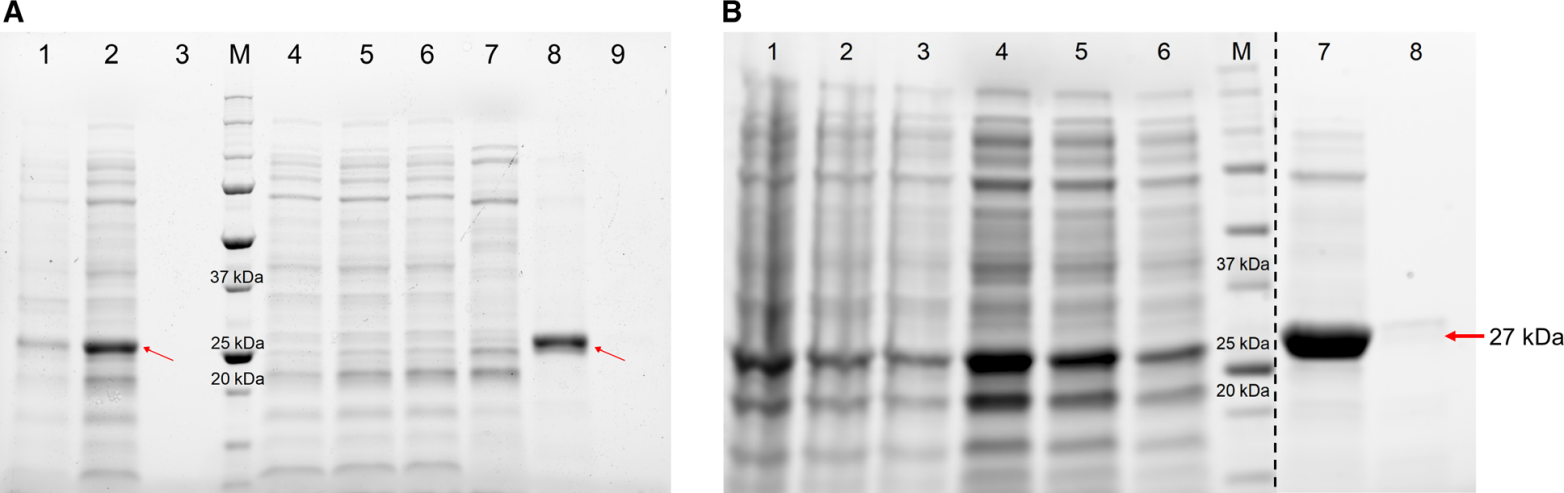
Expression and purification of mutant TEV proteases. (A) The supernatant of cell lysate (lane 2) was separated from the pellet (lane 1) after sonication. Lanes 4–9 indicate the sequential fraction of purification using Ni‐NTA. S219N mutant TEV protease was not eluted from sample injection (lane 4) through the first fraction of 250 mm imidazole buffer (lane 6). Soluble S219N TEV protease was eluted by the second fraction of elution buffer, and its purity was confirmed (lane 8). (B) After sonication, the supernatant of the cell lysate (lanes 4, 5, and 6) was separated from the pellet (lanes 1, 2, and 3). Soluble S219V TEV protease in the supernatant was further purified by the Ni‐NTA column, and the purity of the isolated mutant TEV protease was confirmed (lanes 7 and 8). A dashed line represents a splice mark between two different gels. The size of TEV protease is 27 kDa, as indicated by the red arrow. M represents the protein size marker.

The expression and purity of S219N and S219V mutant TEV proteases with the background of T17S/N68D/I77V mutations were confirmed on SDS/PAGE via a single band with the expected molecular weight of 27 kDa (Fig. 2). The 6xHis‐tagged TEV proteins were overexpressed in the cytosolic fraction and were purified using the Ni‐NTA column and buffer containing 250 mm imidazole. The yield of pure S219V+T17S/N68D/I77V mutant TEV protease was 10 mg·L^−1^ of cell culture, and the yield of S219N+T17S/N68D/I77V mutant TEV protease was 12 mg·L^−1^ of cell culture. The exact concentration of mutant and native TEV protease was measured by the Bradford assay, and their concentrations were almost consistent with the values calculated using an extinction coefficient of 32 220 m ^−1^·cm^−1^.

### Measurement of TEV activity

**Fig. 3:**
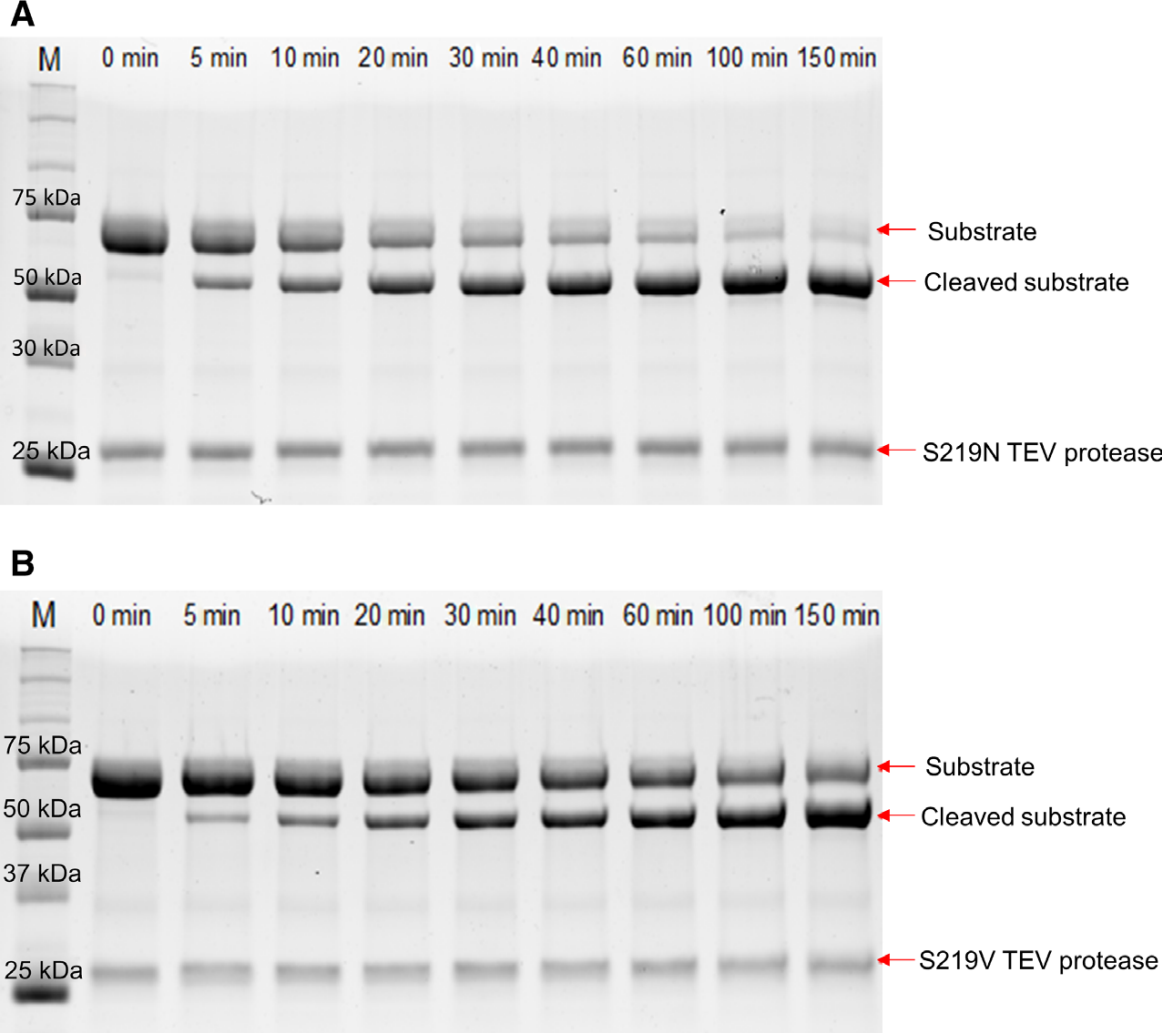
The reaction of TEV protease with RID containing the TEV recognition sequence. (A) SDS/PAGE of the S219N mutant reaction products. (B) SDS/PAGE of the S219V mutant reaction products. The reaction was performed for a total of 150 min, and the reaction samples were collected at the given time points. The size of the substrate RID is approximately 65 kDa, and its size decreased to 50 kDa after cleavage by TEV protease. The size of the protease is 27 kDa, and the band intensity of TEV protease was constant throughout the reaction. The red arrows indicate substrates and TEV protease on the gel. M represents the protein size marker.

The activities of isolated T17S/N68D/I77V+S219V mutant TEV protease and T17S/N68D/I77V+S219N mutant protease were measured via the appearance of the cleaved substrate on SDS/PAGE and fluorescence substrates. Mutant TEV protease (0.1 mg·mL^−1^) was mixed with 65 kDa of recombinant RID fused with norovirus P domain as its substrate containing TEV cleavage sites. As seen in Fig. 3, longer reaction times resulted in an increase in cleaved substrates on the SDS/PAGE, with a constant band intensity of 27 kDa. This result suggests that mutant TEV proteases correctly cleave RID substrates at their cleavage sites and do not cleave TEV protease itself. Interestingly, this cleavage appears to be more efficient when T17S/N68D/I77V+S219N TEV protease was mixed with the substrate rather than T17S/N68D/I77V+S219V mutant TEV protease. After 150 min of incubation with T17S/N68D/I77V+S219N, most of the substrate is cleanly cut into small pieces. It should be noted that a 15‐kDa cleaved substrate product ran off the gel.

**Fig. 4:**
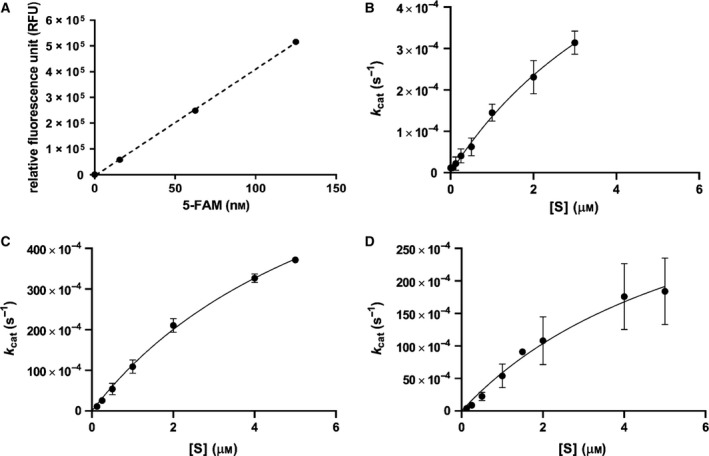
The steady‐state kinetics of TEV proteases. (A) The 5‐FAM standard curve used to convert emission of fluorescence to product concentration. The equation of the standard curve is *y* = 4139.4*x* − 4533 and the *R* ^2^ is 0.9996. (B) The kinetics of control TEV protease, which is one of the components (component C) of the SensoLyte® 520 TEV Protease Assay Kit. (C) The activity of isolated S219N mutant TEV protease. (D) The activity of isolated S219V mutant TEV protease. The data were fitted to the Michaelis–Menten equation, and the error bar represents SD.

The steady‐state kinetics for the reactivity of mutant TEV proteases on substrates fused with 5‐FAM+QXL® 520 quencher was determined. When TEV protease was mixed with the 5‐FAM+QXL® 520 quencher‐linked substrate, TEV recognized the cleavage site Glu‐Asn‐Leu‐Tyr‐Phe‐Gln‐Gly in front of the quencher, and successful cleavage removed 5‐FAM from the quencher, resulting in fluorescence. The concentration of cleaved 5‐FAM was calculated by fitting the released fluorescence to a standard curve of the RFU of 5‐FAM at varying concentrations (Fig. 4A). The emission of fluorescence from the reaction of TEV protease and substrates fused with the 5‐FAM+QXL® 520 quencher was monitored, and then, the initial velocity was calculated in order to plot the increasing concentration of substrates (Fig. 4B–D). The steady‐state kinetics of mutant TEV proteases follows the Michaelis–Menten kinetics (Fig. 4C,D), and the *K*_m_ values were similar to those of the native TEV protease, while the *k*_cat_ values were different (Table 1). The native TEV protease was used as a control from a component of the fluorescence assay kit. There are no published mutations on the control TEV protease. The results indicated that the binding affinity of mutant TEV proteases toward the substrate was similar to that of the native protease, but the *k*_cat_ of the new T17S/N68D/I77V+S219V mutant TEV protease was 50‐fold greater than that of the native TEV protease. Further, the *k*_cat_ of the T17S/N68D/I77V+S219N mutant was twofold greater than that of the T17S/N68D/I77V+S219V mutant TEV protease and 100‐fold greater than that of the native TEV protease. Altogether, we found that the activity of TEV protease was significantly enhanced by mutations on the Ser219 residue with the background of T17S, N68D, and I77V mutations.

**Table 1:** Steady‐state kinetic parameters of substrate cleavage by TEV protease^a^.

	Steady‐state kinetic parameters	
	*K*_m_ (μm)	*k*_cat_ (×10^‐4^ s^−1^)
Control	5.70 ± 2.38	9.07 ± 2.72
S219N	6.82 ± 1.04	884.06 ± 87.46
S219V	6.54 ± 4.02	441.99 ± 175.35

^a^ The steady‐state kinetics of TEV protease was determined by the reaction with a substrate containing 5‐FAM and TEV protease recognition site. The assay was performed using SensoLyte® 520 TEV Protease Assay Kit.

## Discussion

The TEV protease is a useful tool for protein engineering because it truncates substrates at a specific amino acid sequence. However, wild‐type TEV protease has limitations related to its solubility, self‐proteolysis, and cost, which act as hurdles for large‐scale production and usage. In a previous study, many researchers found that variation of Ser219 can both inhibit autoinactivation and improve the activity of TEV protease [18]. It has also been reported that the mutations T17S, N68D, and I77V in TEV protease, which were generated by random mutagenesis, enhance the solubility of the protease [14]. Therefore, we generated S219 mutant TEV proteases on a background of T17S, N68D, and I77V mutations, determined their kinetic parameters using fluorescent substrate, and confirmed their activity on SDS/PAGE.

Thr17, ASN68, and Ile77 residues are located in the loop at the surface of TEV protease. Mutations in these three residues improve the solubility and stability of the protease. According to dynamic simulation, the T17S, N68D, and I77V mutations change the secondary structure pattern, resulting in more α‐helices and β‐sheets that consequently improve the stability of the protease [15]. Using pymol, we confirmed that these residues are located at the surface of the protease (Fig. 1). The protease containing the triple mutation was generated and isolated from the soluble fraction of *E. coli*. The protein yield of isolated S219V mutant TEV protease with the background mutations was 10 mg·L^−1^ of culture media. That of isolated S219N mutant TEV protease with the background mutations was 12 mg·L^−1^ culture media, which is similar to that of the isolated S219V mutant with the background mutations and slightly higher than that of the S219N mutant with no background mutations, as shown in a previous study [14]. In addition, SDS/PAGE showed that the isolated TEV protease clearly truncated its substrate (Fig. 3). These results demonstrated that multiple mutations in TEV protease increased its solubility and productivity, and did not interfere with the ability of TEV protease to recognize its substrate.

**Fig. 5:**
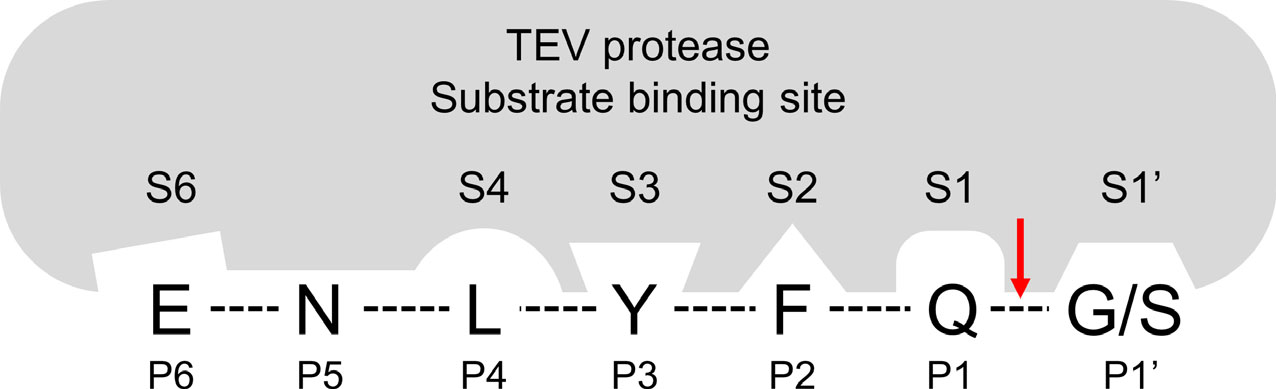
Substrate binding sites of TEV protease. The positions of substrate residues are labeled to P6 to P1′ based on the cleavage site, and S6 to S1′ indicate the corresponding binding pocket in TEV protease. There is no S5 binding pocket in TEV protease because the S5 binding pocket was not formed during substrate binding. The red arrow represents the TEV protease cleavage site, and the dashed lines between residues are the linkage of the substrate residues by peptide bond.

The self‐truncation of wild‐type TEV protease is a critical problem that interferes with its usage. The interaction between the C terminus self‐cleavage site and the active site of TEV protease promotes the modification of the NIa polyprotein [17]. The truncated protease has reduced proteolytic activity, creating difficulties for experimental use. Mutation of the serine 219 residue, especially S219V and S219N, improves the activity of the protease and also inhibits the self‐truncation [18]. In this study, we investigated the inhibition of autoinactivation and the kinetic values of S219 mutants with the background triple mutation. SDS/PAGE results showed that both S219V and S219N mutants had reduced self‐proteolysis while retaining proteolytic activity (Fig. 2). The *K*_m_ value was similar to that of the control, but the *k*_cat_ of both mutants was increased compared to the control (Fig. 4D, Table 1). These results confirmed that Ser219 in TEV protease plays a major role in inhibition of self‐cleavage. Interestingly, the *k*_cat_ of S219N with background mutations was twofold higher than that of S219V, even though the S219V mutant was exhibited 10‐fold more stable activity than the S219N mutant (unpublished result) (Fig. 4, Table 1). It indicates that the activity of the S219N mutant TEV protease was increased by the T17S/N68D/I77V background mutations, which are located at the surface of the protease. When the substrate binds to TEV protease, the protease forms binding pockets based on the substrate’s amino acid sequence [3]. For example, the P1 position of the substrate interacts with the S1 binding pocket in TEV protease, and the P3 position interacts with the S3 pocket of the protease (Fig. 5). The C terminus of TEV protease moves to the vicinity of the active site when the protease recognizes the substrate, and residues 216–218 contribute to the formation of a long and narrow groove, which stabilizes the substrate’s binding through the appropriate positioning of the substrate’s side chains to binding pockets [17]. Residues 217–221 are also involved in the formation of binding pockets S3 to S6, but Ser219 does not come into direct contact with the substrate because its side chain points away from the substrate [8, 19]. This means that Ser219 is located close to the TEV protease active site, but its functional group is exposed to the solvent. In our study, the activity of the newly generated S219N mutant was much greater than that of the S219V mutant with background mutations. It might be explained that the T17S, N68D, and I77V mutations lead to structural changes that improve the substrate binding stability through the effects of asparagine on the positioning of the substrate. The binding pocket may also be expanded by the substitution of a polar amino acid with the nonpolar residue valine, further enhancing the substrate’s accessibility to the active site. Identification of the crystal structure of this quadruple‐mutated TEV protease will be helpful to determine the exact correlation between Asn219 or Val219 in TEV protease and substrate positioning in advance.

In summary, the activity of a newly generated protease was measured in real time using a fluorescent substrate and SDS/PAGE. This allowed us to determine the standard kinetic parameters and to compare them with the control. The result indicates that the multiple mutations given both to improve the yield of TEV protease and to prevent self‐inactivation completely solve the problems at once and even increase TEV activity further.

## Author contributions

MC, DC, and SS conceptualized the study and provided financial support. BH performed cloning of the mutant TEV protease gene and generated the cells containing the mutant gene. HN designed and performed the experiments associated with the kinetics of TEV protease and majorly contributed to writing the manuscript. MC and SS, as the corresponding author, led this research and edited the manuscript. All authors read and approved the final manuscript.

## Conflict of interest

The authors declare no conflict of interest.
